# Prophylaxis with low dose tranexamic acid in acute myeloid leukemia patients undergoing intensive chemotherapy

**DOI:** 10.1002/jha2.699

**Published:** 2023-05-05

**Authors:** Søren Thorgaard Bønløkke, Marianne Tang Severinsen, Hans Beier Ommen, Christian Fenger Eriksen, Anne‐Mette Hvas

**Affiliations:** ^1^ Department of Clinical Biochemistry Aarhus University Hospital Aarhus N Denmark; ^2^ Department of Clinical Medicine Aarhus University Aarhus Denmark; ^3^ Department of Hematology, Clinical Cancer Research Centre Aalborg University Hospital Aalborg Denmark; ^4^ Department of Clinical Medicine Aalborg University Aalborg Denmark; ^5^ Department of Hematology Aarhus University Hospital Aarhus Denmark; ^6^ Department of Anesthesiology Aarhus University Hospital Aarhus Denmark

**Keywords:** acute leukemia, coagulation, fibrinolysis, hemostasis

## Abstract

Patients suffering from acute myeloid leukemia (AML) carry a high risk of serious bleeding complications due to severe thrombocytopenia for long periods of time during treatment. Prior to prophylactic platelet transfusion becoming the standard of care, intracranial bleeding was a major contributor to death in AML patients. However, despite prophylactic platelet transfusions, up to 79% of patients with AML experience clinically significant bleeding during treatment. Antifibrinolytics are effective and well tolerated hemostatic agents widely used in many patient groups, and in this study, we investigated the effect of low dose tranexamic acid (TXA) in patients with AML and thrombocytopenia. We compared bleeding and thrombosis between 113 thrombocytopenic AML patients receiving TXA 500 mg three times daily (*n* = 36) versus no‐TXA (*n* = 77). Clinical information was obtained systematically from electronic medical records, and laboratory data were collected from the laboratory information system. No difference was demonstrated in number of patients with at least one bleeding episode (TXA: 89% vs. no‐TXA: 93%, *p* = 0.60), median number of bleeding days (TXA: 2.5 days vs. no‐TXA 2.0 days, p = 0.30), bleeding location or transfusion needs between the two groups. However, platelet count was found to be a significant risk factor for bleeding, with a probability of bleeding of 35% with a platelet count below 5 × 10^9^/L (logistic regression, *p* < 0.01). We found no difference in thromboembolic events between the two groups (TXA: 8% vs. no‐TXA 10%, *p* = 0.99). In conclusion, treatment with low dose TXA is safe, but we found no evidence to suggest that it reduces bleeding in AML patients with thrombocytopenia.

## 1 INTRODUCTION

Acute myeloid leukemia (AML) is an aggressive cancer developed from hematopoietic stem cells in the bone marrow. Uncontrolled proliferation of immature blasts results in bone marrow failure and subsequent anemia and increased risk of bleeding and infections. Curative treatment of AML includes a series of intensive chemotherapy courses, each leading to severe thrombocytopenia for 2–3 weeks during which, regular platelet transfusions are mandatory to prevent fatal bleeding [1, 2, 3, 4]. Prophylactic platelet transfusions at platelet counts below 10–20 × 10^9^/L are recommended and have reduced but not eliminated the risk of bleeding [2, 4, 5]. Antifibrinolytics such as tranexamic acid (TXA) has been used to reduce bleeding in a number of different patient groups. They are safe and have been proven effective in reducing bleeding in patients undergoing oral surgery and in women with menorrhagia [6, 7, 8]. TXA has also been shown to reduce the need for red blood cell (RBC) transfusions in patients undergoing abdominal surgery [9, 10] and in a study on AML patients by Ben‐Bassat et al. TXA reduced the need for platelet transfusions [11]. However, this study did not include a control group or the use of prophylactic platelet transfusions. Recently, Estcourt et al. reviewed randomized trials regarding the use of antifibrinolytic agents in AML patients [12]. The review concluded that the available evidence was not sufficient to recommend routine use of antifibrinolytics as bleeding prophylaxis in AML patients. However, none of the studies included prophylactic platelet transfusions, which is the current standard of care. Recently, Gernsheimer et al. [13] performed a randomized clinical trial comparing TXA and placebo in 330 patients with various hematological malignancies, of which 151 had AML. They found no difference in bleeding WHO grade 2 or higher between the two groups. However, there was no subgroup analysis for the AML patients, bleeding location was not reported, and the mean treatment time was limited to 14.3 days [13].

Thus, knowledge on efficacy and safety of the use of prophylactic TXA in AML patients with thrombocytopenia is sparse.

## 2 METHODS

We conducted a retrospective cohort study of AML patients treated at Aarhus University Hospital and Aalborg University Hospital, Denmark between 2016–2021. Inclusion criteria were: (1) Diagnosis of AML (ICD‐10 codes: DC920, DC923, DC925‐DC929) (2) age above 18 years, (3) treatment with intensive chemotherapy regimens, and (4) platelet count below 20 × 10^9^/L. Exclusion criteria were acute promyelocytic leukemia.

At both departments, the standard is to follow patients with outpatient visits every other day for red blood cell and platelet transfusions and blood tests during the thrombocytopenic period. Patients treated for AML in Denmark are not admitted during the entire thrombocytopenic period, but rather followed with visits every other day in the outpatient clinic. Prophylactic platelet transfusion is given at platelet counts below 10–15 × 10^9^/L in Aalborg and 15 × 10^9^/L in Aarhus. At Aalborg University Hospital standard clinical practice implies administration of TXA during the thrombocytopenic period if the patient has no history of thromboembolic disease. A low dose of 500 mg three times daily is used. This, however, is not the clinical practice at Aarhus University Hospital, which allowed for the comparison of patients treated at these facilities in regard to the efficacy of TXA prophylaxis.

In some patients at both hospitals TXA was used at higher doses (1000–1500 mg three to four times daily) to treat ongoing bleeding. However, as we wanted to examine the prophylactic effect of low dose TXA (i.e., 500 mg three times per day), data on days where patients received higher doses than 500 mg three times daily were excluded from the analysis.

We collected baseline data at diagnosis including risk factors of bleeding and thrombosis, blood type.

Clinical information, transfusion records, and diagnostic imaging were available in electronic patient records for all patients. Laboratory data were collected from the laboratory information system (LABKA II). Data were collected during the thrombocytopenic period after each course of chemotherapy. We defined the starting point of data collection as the first day with a platelet count below 20 × 10^9^/L and ended data collection when patients sustained a platelet count above 20 × 10^9^/L without platelet transfusion in two consecutive measurements. A level of 20 × 10^9^/L was chosen because some patients received platelet transfusions at platelet counts between 15–20 × 10^9^/L. This was done for each course of induction and consolidation chemotherapy. We recorded results of hemoglobin and platelet count every time it was measured during the thrombocytopenic period. Other coagulation tests were not systematically measured, and thus not reported here. Clinical information included bleeding and thrombosis, whether the patients received any transfusions and the current dose of TXA. The location and severity of bleeding were recorded. All thromboembolic events were registered from treatment start to the end of data collection. If a patient suffered from a thrombosis, data after the event were excluded due to treatment with anticoagulants and increased platelet transfusion limits.

All days where bleeding was registered counted toward number of bleeding days. However, each patient was only counted once per bleeding location in the bleeding location analysis.

## 3 STATISTICAL ANALYSIS

Descriptive statistics are presented as mean and standard deviation for normally distributed data and as median and interquartile range for non‐normal distributed data. For comparisons of categorical values between groups Fisher's exact test was used, and for continuous variables Wilcoxon rank sum test was used. Association between platelet count, infection, and bleeding risk was evaluated using logistic regression with platelet count interval (<5, 5–10, 10–15, >15 × 10^9^/L) and infection (yes/no) as predictors of bleeding. The model was adjusted for repeated measurements by clustering by patient id. Using the glht package and the mcp function, a multiple comparison between the coefficient were performed. Statistical analysis was performed using RStudio 2022.02.2 + 485 “Prairie Trillium” Release (8acbd38b0d4ca3c86c570cf4112a8180c48cc6fb, 2022‐04‐19) for Windows.

## 4 RESULTS

We identified 113 patients and collected data on 2696 days, where patients were dependent on platelet transfusions to keep the platelet count above 10–20 × 10^9^/L. Of the 113 patients, 36 (32%) patients received prophylactic TXA at a dose of 500 mg three times per day (the TXA‐group) whereas 77 (68%) patients did not receive TXA (the no‐TXA‐group). On 210 days the TXA dose was higher than 500 mg three times daily, and data from these days were excluded from analysis. No difference was observed regarding demographic or clinical characteristics between the two groups (Table 1). There was no difference in the platelet count on days where patients received platelet transfusions (10 × 10^9^/L vs. 11 × 10^9^/L, *p* = 0.08).

**TABLE 1:** Demographic and clinical characteristics, bleeding, biochemistry, and transfusions among 113 patients with acute myeloid leukemia.

Baseline	All	TXA	No‐TXA	
	*n* = 113	*n* = 36	*n* = 77	*p*‐value
Median age, y (IQR)	59 (49–67)	59 (48–66)	59 (49–67)	0.92
Male, *n* (%)	58 (51)	14 (39)	44 (57)	0.11
Median BMI, kg/m2 (IQR)	27 (23–30)	26 (23–29)	27 (24–31)	0.21
Smoking, *n* (%)	60 (53)	18 (49)	23 (40)	0.30
Previous thrombosis, *n* (%)	2	1 (3)	1 (1)	0.54
Venous, *n*	1	0	1	
Arterial, *n*	1	1	0	
Type 2 diabetes, *n* (%)	5 (4)	2 (6)	3 (4)	0.65
Blood type 0, *n* (%)	50 (44)	18 (50)	45 (58)	0.42
**Bleeding**				
Patients with any bleeding during follow‐up, *n* (%)	96 (85)	32 (89)	64 (83)	0.60
Bleeding days, median (IQR)	2 (1–5)	2.5 (1–6)	2 (1–4)	0.30
Thrombocytopenic days, median (IQR)	37 (23–49)	38 (21–50)	35 (25–48)	0.99
**Biochemistry during follow up**				
Platelet count 10^9^/L, median (IQR)*	13 (9–18)	12 (8–16)	13 (9–19)	
Hemoglobin mmol/L, median (IQR)*	5.4 (5–5.8)	5.5 (5.2–5.9)	5.4 (5–5.8)	
**Transfusions**				
RBC transfusions, median (IQR)	10 (7–16)	11 (7–14)	10 (7–17)	
Platelet transfusions, median (IQR)	16 (11–25)	20 (8–28)	16 (11–21)	

*Note*: Patient characteristics, bleeding, biochemistry, and transfusions. *p*‐values are for comparison of the TXA versus no‐TXA groups. Abbreviations: BMI, body mass index, RBC, red blood cell.

As shown in Table 1, we found no difference in the proportion of patients experiencing at least one bleeding episode during the thrombocytopenic periods between the two groups. Additionally, there was no difference in the number of RBC and platelet transfusions between the two groups. Six patients in the no‐TXA group received fresh frozen plasma (FFP) transfusions due to prolonged bleeding: two due to hematuria, two due to menorrhagia, one due to epistaxis and one due to gastrointestinal bleeding. No patients in the TXA group received FFP in relation to bleeding, however one patient in TXA group received FFP prior to replacing a central venous catheter.

As shown in Table 2, the TXA‐group experienced numerically fewer mucosal bleeds (odds ratio [OR]: 0.59; 95% confidence interval (CI): 0.24–1.41, *p* = 0.23) and skin bleeds (OR: 0.76; 95% CI: 0.30–1.92, *p* = 0.52) than the no‐TXA‐group. However, the differences were not statistically significant.

**TABLE 2:** Number of acute myeloid leukemia patients with at least on bleeding episode in each location.

	TXA (*n* = 36)	No‐TXA (*n* = 77)	*p*‐Value
Skin, *n* (%)	23 (70)	54 (64)	0.52
Mucosal, *n* (%)	14 (39)	40 (52)	0.23
Gastrointestinal, *n* (%)	2 (7)	5 (6)	0.99
Hematuria, *n* (%)	3 (8)	6 (8)	0.99

Eleven patients suffered from venous thromboembolic events (VTE). No arterial thromboses were observed. Of the 11 thromboses, six were related to central venous catheters, three events occurred in lower limb deep veins, and two were peripheral pulmonary embolisms. One patient had prior history of VTE previous to a cancer diagnosis. Three out of the 11 patients received prophylactic TXA when the thrombosis was diagnosed, all having catheter‐associated deep vein thrombosis. Four additional patients had received TXA but the treatment had stopped 10–23 days prior to the thrombosis. The remaining four patients had not received TXA.

**FIGURE 1:**
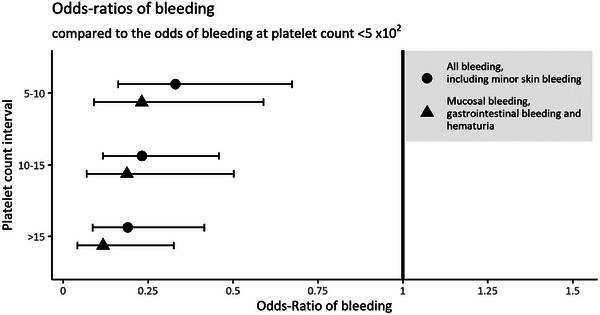
Odds‐ratios of bleeding compared to the odds of bleeding at platelet count <5 × 10^9^/L. Odds ratio of bleeding decreases with increases to platelet count within the defined intervals (5–10, 10–15, and >15 × 10^9^/L).

In order to analyze the association between platelet count and bleeding we grouped platelet counts in four groups (<5, 5–10, 10–15, and >15 × 10^9^/L) and analyzed the association as described in the statistical analysis section. The probability of bleeding in the <5 × 10^9^/L group was 0.35, *p* < 0.01. If skin bleeding was excluded the risk of bleeding in the <5 × 10^9^/L was 0.22, *p* < 0.001. As shown in Figure 1, the odds‐ratio of bleeding is significantly reduced with rising platelet count. We compared the <5 group with the rest using a Wald‐test yielding a *p*‐value ≤ 0.001, showing a significant difference in bleeding risk between platelet count <5 and the other groups.

## 5 DISCUSSION

This is the first study to evaluate the benefit of prophylactic TXA in AML patients with severe thrombocytopenia, whom received prophylactic platelet transfusion in concordance with current guidelines [4]. We demonstrated this low dose TXA to be safe, but it offered no reduction in clinically significant bleeding.

The dose of TXA administered in this study was low compared to other studies where no platelet prophylaxis was given in order to avoid excessive thrombosis risk [11, 14]. Our data show that the present low dose TXA is safe as we demonstrated no evidence of increased thromboembolic risk. Although more patients in the TXA group developed VTE, four of seven patients had discontinued TXA more than a week prior to the VTE diagnosis. The low half time of TXA in plasma (3 h) makes it implausible that administration of TXA is the cause of VTE in these patients. This is also supported by available evidence showing that TXA at higher doses does not constitute an increased VTE risk in other patients [13, 15]. Based on this study it is not possible to conclude whether the lack of effect of TXA is due to insufficient dosage or the lack of fibrinolysis in the population.

Currently two ongoing trials TREATT [16] (1 gram intravenously / 1.5 g orally every 8 h) and PATH [17] (1 g every 8 h) evaluate the use of prophylactic TXA in various severely thrombocytopenic hematological patients. Use of this higher dose might show beneficial effect; however, this still needs clarification.

We have showed that a very low platelet count is associated with increased risk of bleeding, but there seems to be no difference in risk between platelet counts of 10–15 × 10^9^/L and > 15 × 10^9^/L in this study. This is well in line with the current practice of administering prophylactic platelet transfusions when platelet count drops below 10–15 × 10^9^/L. Infections did not have an effect on bleeding risk in our analysis, which indicates that there is no reason to have different transfusion limits for patients who are infected.

The present study is limited by the retrospective collection of data that data were collected by only one person, data collection was not blinded, and that exposure and outcome data were collected from the same source. The difference in platelet transfusion threshold could lead to less bleeding in the no‐TXA group, but there was no difference in the platelet count between groups on the days where platelet transfusions were administered. The study is strengthened by its relatively large study population considering the rarity of AML. Moreover, the TXA and no TXA‐group were similar. Further, data were collected systematically, and variables were defined and validated prior to the final data collection.

## 6 CONCLUSIONS

In conclusion, TXA 500 mg three times daily can safely be given to AML patients in thrombocytopenia after chemotherapeutic treatment, but this prophylaxis does not seem to reduce bleeding risk.

## AUTHOR CONTRIBUTIONS

STB, HBO, CFE, MTS, and AMH conceived and formulated the study. STB collected and analyzed the data and wrote the first draft of the manuscript. All authors critically revised the manuscript.

## CONFLICT OF INTEREST STATEMENT

STB has no conflict regarding the present work but has received unrestricted research support from CSL Behring. AMH has no conflict of interest regarding the present work but has the following general conflict of interest: Unrestricted research support from CSL Behring. Other authors have no conflict of interest.

## FUNDING INFORMATION

The authors received no specific funding for this work.

## ETHICS STATEMENT

Study was done as a quality control study and thus was approved by the Hospital Director at either hospital prior to initiation. The authors have confirmed patient consent statement is not needed for this submission. The authors have confirmed clinical trial registration is not needed for this submission.

## Data Availability

The data that support the findings of this study are available from the corresponding author upon reasonable request.
